# Airborne Microorganism Inactivation by a UV-C LED and Ionizer-Based Continuous Sanitation Air (CSA) System in Train Environments

**DOI:** 10.3390/ijerph19031559

**Published:** 2022-01-29

**Authors:** Giulia Baldelli, Mattia Paolo Aliano, Giulia Amagliani, Mauro Magnani, Giorgio Brandi, Carmelo Pennino, Giuditta Fiorella Schiavano

**Affiliations:** 1 Department of Biomolecular Sciences, University of Urbino Carlo Bo, 61029 Urbino, Italy; giulia.baldelli@uniurb.it (G.B.); giulia.amagliani@uniurb.it (G.A.); mauro.magnani@uniurb.it (M.M.); giorgio.brandi@uniurb.it (G.B.); 2 STE—Sanitizing Technologies and Equipments s.r.l., 61020 Petriano, Italy; aliano@stesanitizing.com; 3 AF Frigo Clima Impianti S.r.l., 41030 Bomporto, Italy; pennino@af-frigoclimaimpianti.it; 4 Department of Humanities, University of Urbino Carlo Bo, 61029 Urbino, Italy

**Keywords:** air sanitation, public transportation, heating, ventilation and air conditioning (HVAC), UV-C LED, SARS-CoV-2

## Abstract

Improving indoor air quality present in environments where people live is important to protect human health. This particularly applies to public transportation, where air quality may affect the health and safety of passengers, workers and staff. To provide better air quality, many buildings and transports are provided with heating, ventilation and air conditioning (HVAC) systems, which are always equipped with filters to retain the particulate present in the airflow, but they lack continuous air sanitization systems. In this study, a new UV-C LED and ionizer-based continuous sanitation air (CSA) system to be installed in a train HVAC was developed (international patent: N.PCT/IB2021/054194) and its sanitation efficacy against various microbial species (bacteria and fungi) was assessed. The device proved to be very effective at the microbial killing of aerodispersed microorganisms, both in its experimental configuration (ISO 15714:2019) and in a train setting. The installation of this CSA system on public transportation appears to be a promising solution to guarantee high microbiological air quality with a very low environmental impact due to its eco-friendly components.

## 1. Introduction

The hygienic quality of the air and surfaces of indoor environments can affect the health of people living and working in them due the presence of different microorganisms, physical and chemical factors, pollutants, and particulate matter [1]. Eight major sources of airborne microbiotes found in indoor environments have been identified, including: humans; pets; plants; plumbing systems; heating, ventilation, and air-conditioning (HVAC) systems; mold; dust resuspension; and the outdoor environment [2]. Moreover, the type, survival and spread of airborne microorganisms are also tightly linked to physically favorable environmental parameters, such as relative humidity (RH, %) and temperature (T, °C) [3]. The daily air inhaled by a human typically contains average loads of 10^6^ airborne microorganisms [4], some of which can be pathogenic and cause illnesses of different severity, from flu to COVID-19, pneumonia, asthma, or allergies [5,6,7,8].

Indoor airborne microbial content is detected in variable concentrations, depending on seasonal changes and on the considered environment [9,10,11]. Microbial loads of 10^2^–10^3^ CFU/m^3^ have been detected in public transportation environments [12,13]. Particular attention must be paid to such at-risk indoor settings because they can be crowded environments in which people coming from different places can stand for a long time together, being exposed to several aerodispersed microbial species. Most importantly, it must be considered that in this type of indoor environment, the air is mainly recycled, without the possibility of renewal [14]. Moreover, the mean respirable fractions for bacteria and fungi detected in train cars is much higher than in other indoor environments (62.8% and 81.4% vs. 32.0–38.1% and 58.9–69.1% for bacteria and fungi, respectively) [10,12], resulting in higher adverse health risks for sensitive commuters and workers. To provide better air quality, in the last decades, many buildings and transport vehicles were equipped with HVAC systems. In railway vehicles, air conditioning systems are generally air-handling units that cool or heat the air according to the period of the year. To minimize energy consumption and power, and to attenuate the increase in CO_2_ values due to the presence of people on board, these systems treat the indoor air with continuous recirculation. Moreover, all HVAC systems are equipped with filters to retain the particulate present in the airflow, but they lack a continuous air sanitization system. Therefore, efficient sanitation devices are needed to improve environmental microbiological quality in transportation. Several methods have been tested to sanitize indoor air [15,16,17,18,19,20]. These strategies are based on different technologies, such as filtration, ultraviolet C (UV-C) light, ionizing radiation, liquid, and gaseous chemicals, although some can be harmful to human health or produce unsafe by-products. One of the most deeply studied methods of indoor environment sanitization is UV-C LED light with a wavelength of 200–280 nm. UV-C lamps for ultraviolet germicidal irradiation (UVGI) installed in HVAC ducts have been demonstrated to efficiently inactivate airborne pathogens [21,22]. However, the effectiveness of UV disinfection depends on several factors, such as the power of the UV source, the exposure time, the distance of microorganisms from the source, microorganism resistance, the presence of particulate and shadow zones, etc. Airborne viruses, such as influenza virus, and SARS-CoV-2, are more easily inactivated by UV-C than other microorganisms and non-enveloped viruses [23]. At a virus density comparable to that observed in SARS-CoV-2 infection, a UV-C dose of just 3.7 mJ/cm^2^ was sufficient to achieve a more than 3 log inactivation without any sign of viral replication [24]. A certain variability has been observed among bacterial species. By contrast, spores and filamentous fungi are the most difficult to penetrate and inactivate with UV-C and require higher UV-C doses [25,26]. The COVID-19 pandemic highlighted the need to ensure a high level of microbial air quality in indoor environments. This particularly applies to public transportation, where air sanitation devices could be essential to safeguard the health and safety of passengers, workers and staff [14]. However, no scientific data about sanitizing systems on trains have yet been reported. The aim of this study was therefore to evaluate the air sanitation efficacy of a UV-C LED and ionizer-based continuous sanitation air (CSA) system to be installed in a train HVAC. The system’s sanitation efficacy against various microbial species (bacteria and fungi) and at different airflow rates was assessed both in an experimental configuration (test rig) and in a train setting.

## 2. Materials and Methods

### 2.1. Continuous Sanitation Air (CSA) System

**Figure 1:**
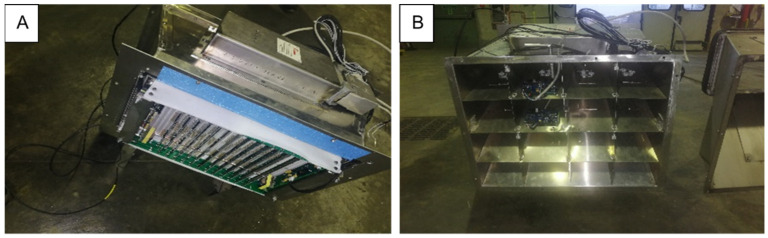
Front view (**A**) and sub-ducts posterior view (**B**) of CSA system.

The CSA system is a continuously operating air sanitation and purification system that aims at killing airborne microorganisms and reducing potential pollutants in the air (e.g. PM1–10). The CSA system, which was developed for railway applications, was installed outside the passenger compartment, inside the HVAC box, and/or inside the ventilation ducts in an accessible position. The system (international patent application: N.PCT/IB2021/054194) consists of an ionizer, a ISO Coarse 90% self-sanitizing filter [27], UV-C LEDs, a sensor platform for detecting air quality parameters and an electronic control Unit (ECU). The CSA system (Figure 1) is made of a metallic main duct that holds the ionizer and the filter in the first portion. After the filter, the main duct is split into sub-portions that divide the airflow in the sub-conducts. The UV-C LEDs are arranged in these sub-ducts to sanitize the airflow and the internal surface of the filter; moreover, some other LEDs are placed on the ionizer to sanitize the external filter surface. The sensor platform, placed at the end of the duct, make it possible to continuously check the air quality parameters such as pressure, airflow speed, temperature, relative humidity, atmospheric particulate matter (PM10, PM4, PM2), and gaseous pollutants (VOC, CO_2_, O_3_, H_2_S). 

**Figure 2:**
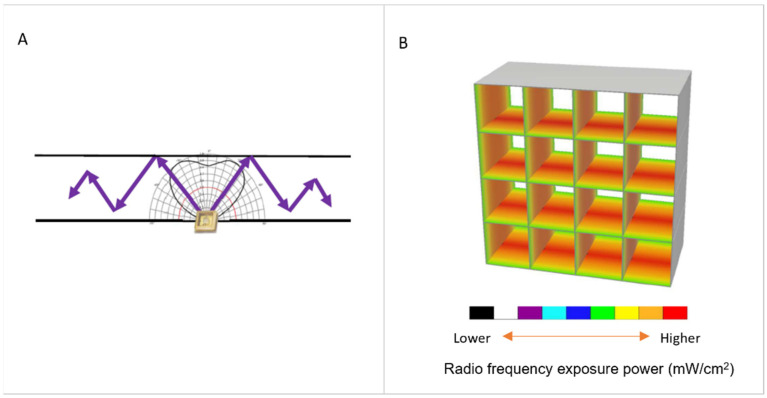
(**A**) LED UV-C ray reflections; (**B**) simulation of LED UV-C ray reflections inside sub-ducts. The color scale is representative of the radio frequency exposure power (mW/cm^2^) and to the refraction.

The UV-C LEDs produce light rays with a specific wavelength (275 ± 5 nm) starting from small amounts of electricity (in the order of a few mWs) with a low thermal resistance. To maximize UV-C radiation, the sub-ducts were designed with particular geometries and special surface treatments that increase the number of UV-C ray reflections in the air path (Figure 2). 

### 2.2. Test Microorganism Suspension Preparation

*Escherichia coli* (ATCC 25922), *Bacillus subtilis* (ATCC 6633) and *Cladosporium* spp. were selected as test microorganisms, in accordance with the ISO 15714:2019 standard [26]. *Bacillus subtilis* (ATCC 6633) and *Cladosporium* spp. were selected in accordance with the ISO 15714:2019 standard [26]. The same standard also included *Serratia marcescens*. However, instead of this species, the microorganism *Escherichia coli* was used as it belongs to the same family (Enterobacteriaceae) and shows a similar UV-C susceptibility (as reported in the ISO 15714:2019, Annex C), but entails a lower biological risk for operators. *E. coli* was grown in tryptic soy agar (TSA) plates supplemented with 0.05 g/L cycloheximide and *B. subtilis* (vegetative form) in brain heart infusion (BHI) agar, both at 32 °C. Overnight cultures of bacterial strains in tryptic soy broth (TSB) at 32 °C were washed and resuspended in saline solution (NaCl 0.9%), then adjusted at optical densities at 600 nm (OD_600_), corresponding to a final concentration of 10^8^ (*E. coli*) and 10^7^ (*B. subtilis*) CFU/mL. *Cladosporium* spp. was grown in agar slant tubes containing malt extract agar (MEA) with 0.05 g/L chloramphenicol at 25 °C for at least 72 h. The fungal suspension was prepared pouring saline solution over *Cladosporium* spp. slants to gently swirl and detach fungal conidia. The fungal suspension was then diluted to optical density at 530 nm (OD_530_) to a final concentration of 10^6^ CFU/mL. The microorganism suspensions’ concentrations were experimentally confirmed by plate culture. All culture materials were purchased from Thermo Fisher Scientific (Waltham, MA USA).

### 2.3. Test Rig Configuration

**Figure 3:**
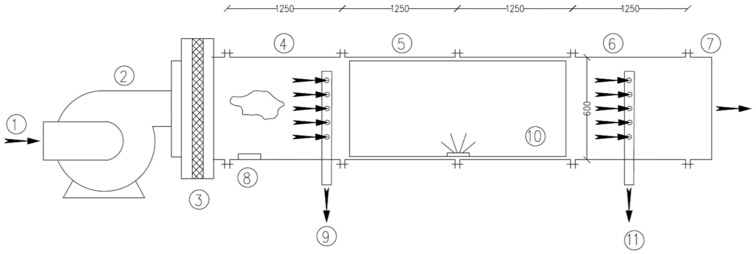
Test rig configuration. ① Air-intake/air inlet/inflow/entrance; ② variable flow rate blower; ③ HEPA filter; ④ upstream duct; ⑤ UVGI device mounting duct; ⑥ downstream duct; ⑦ off-gas pipe; ⑧ nebulizer; ⑨ upstream microorganism sampling port; ⑩ CSA system; ⑪ downstream microorganism sampling port.

A test rig was built in compliance with the standard for the experimental testing procedures (Figure 3) [26]. It included: (a) a variable flow rate blower, used to control the flow rate of the test system, installed in front of the test rig; (b) a HEPA filter placed before the upstream duct to remove potentially contaminating airborne microorganisms present in the air, in order to prevent their impact on test microorganism quantification; (c) an upstream duct equipped with a nebulizer connected to a test microorganism injection port less than 20 mm in diameter, set near the left flange of the duct; (d) the CSA system mounting duct; (e) a downstream duct with a sampling port for downstream sampling, set near the right flange of the duct; (f) an off-gas pipe. The ducts were made of aluminum and had a square cross-section and an inner side length of 600 mm. The individual ducts had a length of 1250 mm each and were connected and sealed by an insulating material, to avoid air leakage during the experiment. Inside each sampling port, a perforated cylindric tube (110 mm in diameter, with five holes of 20 mm in diameter) was placed to accommodate the head of the air sampler. The sampling ports were set to the central line of the lower wall and were closed during the test. The air outflow was funneled in a chamber with a single outtake opening and a HEPA filter of 110 m^3^ in volume. Inside this chamber, a digitally controlled thermic nebulizer, equipped with an HP3 germicide, was placed.

### 2.4. Air Sampling

The air was sampled with a Surface Air System sampler (SAS, PBI International, Milan, Italy), collecting air for 1 min, with an air volume of 180 L impacting the agar plates (TSA 0.05 g/L cycloheximide, BHI agar and MEA 0.05 g/L chloramphenicol). For each test, the air was sampled three times. After the air samplings, the agar plates were transferred to the laboratory in refrigerated conditions, then incubated at 32 °C for 24–48 h (*E. coli* and *B. subtilis*) or 25 °C for 72–120 h (*Cladosporium* spp.). The colony forming unit per cubic meter of air (CFU/m^3^) was then calculated using the following formula:

(1) $$\mathrm{CFU}/m^{3}=\frac{\mathrm{number}\;\mathrm{of}\;\mathrm{colonies}\;\mathrm{in}\;\mathrm{each}\;\mathrm{plate}}{\mathrm{air}\;\mathrm{volume}\;\mathrm{sampled}\;\left(l\right)}\times 1000$$

### 2.5. Assessment of CSA System Sanitation Efficiency in the Test Rig

The assessment of the performance of the CSA system was carried out according to the standard [26]. A liquid nebulizer for suspensions (Collison nebulizer, AGK 2000 Palas GmbH, Karlsruhe, Germany) was used to generate an aerosol of the test microorganisms, upstream of the CSA system. The nebulizer was connected to a compressor equipped with particulate, water and oil repellent filters. The pressure was set at 3 atm throughout the tests. The nebulization was maintained for 2 min for each test, which was the time required to nebulize 1 ml of the microbial suspension. To achieve a correct initial microbial dispersion, during the aerosolization of the microorganisms, the airflow rate was maintained at 320 m^3^/h. The disinfection efficacy of the CSA system was tested at three different airflow rates: 1000, 2000 and 3000 m^3^/h. The air was sampled upstream and downstream of the CSA system; the downstream samples were obtained with the sanitation system switched off and switched on to calculate the sanitation efficiency. Moreover, the contribution of each CSA system component (UV-C LED, Electrostatic Space Charge System (ESCS) ionizer and ISO Coarse 90% filter) to the induction of the air sanitation was separately considered in the test rig configuration, nebulizing *E. coli* suspension, with a 3000 m^3^/h airflow rate. For this purpose, the experiment was repeated by switching on one element at a time or removing/installing the filter. Temperature (°C) and relative humidity (% RH) were registered with the validated sensor of the CSA system before and after each experimental session. 

### 2.6. Assessment of CSA System Sanitation Efficiency in the Train Setting

The assessment of the performance of the CSA system was subsequently carried out in a Vivalto train setting.

*Background microbial load*. Before each experiment, an initial air sample from the train car (in a median position in the coach and in the proximity of the air vents) was obtained to determine the environmental bacteria (TSA) and fungi (Potato Dextrose Agar) concentrations.

**Figure 4:**
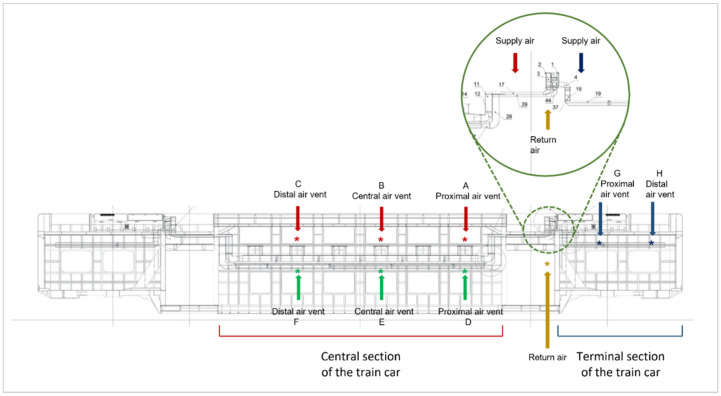
Experimental setting in the Vivalto train. Sampling sites: proximal (A), central (B) and distal (C) air vent panels of the upper central section of the train car (red arrows); the proximal (D), central (E) and distal (F) air vent panels of the lower central section of the train car (green arrows); the proximal (G) and distal (H) air vent panels in the terminal section of the train car (blue arrows). Point of determination of *E. coli* starting concentration after nebulization (yellow).

*Inactivation test*. The nebulization step was performed as previously described. To produce a dispersed aerosol of *E. coli* and to reach the desired concentration of aerosolized microorganism, a nebulization pre-chamber was built and located in the vicinity of the train car return air panels. After nebulizing the suspension for 2 min, the aerosolized microorganisms were aspirated by the conditioning system and passed through the CSA system. The air was sampled in the vicinity of the train car supply air panels, with the airflow switched on. To obtain results as representative as possible of the entire train car, eight different air vents were selected as sampling points: the proximal, centra, and distal air vent panels of the upper central section of the train car; the proximal, central and distal air vent panels of the lower central section of the train car; and the proximal and distal air vent panels in the terminal section of the train car (Figure 4). In the Vivalto air handling unit, the airflow rate was 4000 m^3^/h. The airflow rate overflowing from the air vent panels was measured with an anemometer at each sampling point considered. Air samples at the nebulization pre-chamber were also obtained The experiments were carried out in the train car without the CSA system; next, the disinfection device was placed in the air conditioning system and the tests were performed with the CSA system switched on. Temperature and relative humidity were registered as described above.

### 2.7. Inactivation Rate

The air disinfection efficiency of CSA system was expressed as inactivation rate (% IR), applying the following formula:

(2) $$\%\mathrm{IR}=\frac{\left(A-B\right)}{A}\times 100$$

in which A is the microbial concentration (CFU/m^3^) sampled downstream of the CSA system with the disinfection device switched off and the ISO Coarse 90% filter removed, while B is the microbial concentration (CFU/m^3^) sampled downstream of the CSA system with the disinfection device switched on and ISO Coarse 90% filter installed.

### 2.8. Determination of UV-C Dose Required for the Inactivation of the Microbial Species

The UV dose needed for the inactivation of the microbial species used in this study can be determined by the inactivation rate and the microbial susceptibility (*k*). The UV dose was determined according to the standard [26]:

(3) $$\ln\left(\frac{N_{0}}{N}\right)=kD$$

where *N*_0_ is the original active microorganism concentration, *N* is the active microorganism concentration after disinfection, D is the UV dose and *k* is the susceptibility constant. *k* was obtained from Annex C of the standard and it was 0.38 m^2^/J for *E. coli* (average of different studies), 0.16858 m^2^/J for *B. subtilis* and 0.0021 for *Cladosporium* spp. [26].

## 3. Results

### 3.1. Microclimatic Parameters 

The experiments were carried out over periods of different months. The temperature and % RH registered before and after each experimental session were included in a range of 18–33 °C and 42–63% RH. In the train setting, the microclimatic parameters ranged as follows: 19–24 °C and 43–57% RH. The duplicate assessment of each parameter indicated that the relative difference in the same index was less than 10%, ensuring the stability of the testing system. However, the relative variations of the same parameters at the beginning and at the end of the experimental session were occasionally higher than 10%.

### 3.2. Microbial Inactivation Rates in the Test Rig Configuration

The air samples taken upstream of the CSA system in the test rig were useful to check the concentration of microorganisms aerosolized during the tests. These concentrations, after a 2 min aerosolization during which an airflow rate of 320 m^3^/h was maintained, were the following: 7.5 ± 0.17 *×* 10^3^ CFU/m^3^ (*E. coli*); 1.91 ± 0.13 × 10^3^ CFU/m^3^ (*B. subtilis*); 1.09 ± 0.02 × 10^3^ CFU/m^3^ (*Cladosporium* spp.). The microbial concentrations of the aerodispersed microorganisms were also measured downstream of the CSA system, with the sanitation system switched off and the ISO Coarse 90% filter removed, or with the sanitation system switched on and the ISO Coarse 90% filter installed. Inactivation percentages of the bacterial species, *E. coli* and *B. subtilis*, were always very high and ranged from 98.96 to 100%. Lower % IR were instead obtained for *Cladosporium* spp. (67.88–69.45%) (Table 1). These data showed a mean reduction of 2 log for aerosolized *E. coli* and 2–3 log for aerosolized *B. subtilis*. For all tested microorganisms, the % IR were not affected by the three different airflow rates used in this study (Table 1).

### 3.3. Assessment of Component Contribution on Microbial Inactivation Rates in the Test Rig Configuration

**Figure 5:**
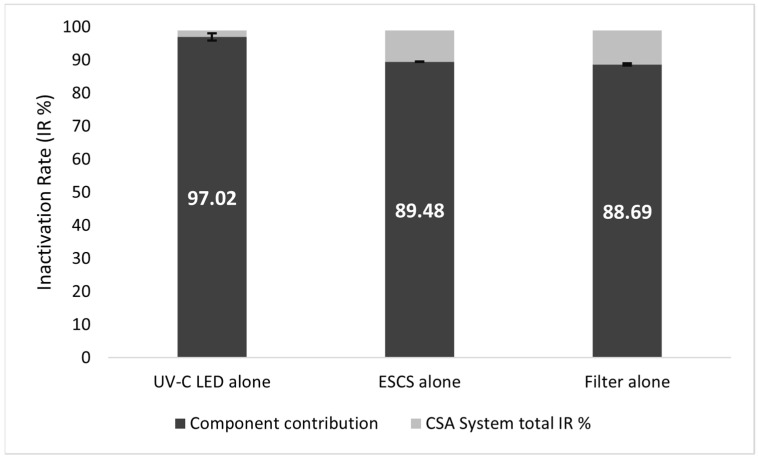
Contribution of each CSA system component to air sanitation efficiency in the test rig configuration.

The assessment of each CSA system component (UV-C LED, ESCS ionizer and ISO Coarse 90% filter) contribution to the sanitization of the air from *E. coli*, with a 3000 m^3^/h airflow rate, showed that the greatest % IR was achieved by the UV-C LED, leading to a 97.02 ± 1.06% efficiency, in comparison to the 98.96 ± 0.6% achieved by the entire CSA system. On the other hand, the ESCS or the ISO Coarse 90% filter alone led to lower sanitation efficiency (89.48 ± 0.08% and 88.69 ± 0.37%, respectively) (Figure 5).

### 3.4. Microbial Inactivation Rates in the Train Setting

The background microbial load was always around 50–200 CFU/m^3^ (bacteria) and 100 CFU/m^3^ (fungi). Near the air vents, a microbial load around 50 CFU/m^3^ (bacteria) and 80 CFU/m^3^ (fungi) was recorded. The airflow rate overflowing from the air vent panels, measured with an anemometer at each sampling point considered, ranged from 0.25 to 0.54 m/s in the upper central section; 0.21 to 0.58 m/s in the lower central section; and 0.87 to 1.02 m/s in the terminal section of the train car. The concentration of the *E. coli* aerosolized during the tests was 1.19 ± 0.45 × 10^5^ CFU/m^3^. The inactivation rates of the different sampling sites (Table 2) were, on average, 95.45 ± 1.46%, 92.69 ± 3.00% and 93.12 ± 1.19% in the upper, lower and terminal sections of the train car, respectively.

### 3.5. Determination of UV Dose Required for the Inactivation of the Microbial Species

The UV doses for each microorganism and at each airflow rate are reported in Table 3. In the train setting, the UV doses ranged from 3.99 to 10.32 J/m^2^. 

## 4. Discussion

Air sanitation in crowded environments, such as in public transportation, is of paramount importance to provide healthy and safe travel conditions to passengers and workers. In this study, a new air sanitation device, consisting of multiple components with a sanitizing effect (UV-C, filter and ionizer), was designed to be installed in an HVAC system and tested for its efficiency at microbial inactivation. The aerodispersed microorganism UV-C susceptibility strongly depends on different factors, including the ability of different microbial species to recover UV radiation-induced damage, the UV dose, irradiance, RH, exposure time, and the particulate/moisture present in the air [25,28]. As required by the standard, three microorganisms with different UV-C susceptibility levels were chosen [26]. The first experiments aimed to calculate the percentage of IR of the CSA system for each microorganism in the test rig configuration [26]. The results highlighted the optimal inactivation efficiency with bacterial species (*E. coli* and *B. subtilis*) at all the airflow rates tested. A minor effect with filamentous fungi (*Cladosporium* spp.), known to be more resistant to UV-C, was instead noticed [26,29].

The estimated UV-C dose (D) required for the inactivation of each specific microorganism depends on different factors: the airflow rate, the microbial susceptibility, and the IR. Indeed, the longer a microbe is exposed to UV-C light, the higher the UV-C dose it will receive; therefore, D is inversely proportional to the airflow rate (1000, 2000, 3000 m^3^/h) used in the test rig. Moreover, the UV-C LED sanitation efficacy is inversely proportional to the distance from the UV-C LED source [30]. In the proposed device, the UV-C dose led to an optimal inactivation rate even with a high airflow rate, of 4000 m^3^/h. This result was due to the patented structure of the sub-ducts, which made it possible to subdivide the airflow, thus reducing the airflow rate, and to decrease the distance of the UV-C source. Considering the IR/retention separately provided by each different component of the CSA system, a prevalent effect of the UV-C was deduced. Our results allowed us to exclude that the filter or the ionizer alone may have a sufficient efficacy to provide optimal levels of microbial inactivation or retention in order to ensure air sanitation. According to previous results, the link between the inactivation of microorganisms and the ionizer seems to be mainly related to the ozone produced and not to the electroporation itself, suggesting that the bactericidal action attributed to ionizers may have been overestimated [31]. In the train setting, a very high IR was confirmed with *E. coli*. Besides the microbial susceptibility and flow rate, the performance of an in-duct sanitation device can be affected by temperature and RH, although microclimatic parameters have been considered less important [26]. In our experiments, these parameters were sometimes slightly different from those indicated as optimal in the standard method (25 ± 2.5 °C and 50 ± 10%) [26], especially at the beginning of the experimental session, and reflected seasonal variations in climate. Moreover, it is important to highlight that an inverse relationship exists between RH, temperature, and the efficacy of UV devices, with a lower IR result at higher RH and lower temperature [28,32]. Therefore, the initial RH of lower than 40% and the temperature higher than 27.5 °C assessed in some experimental sessions could have partially decreased the IR of the sanitation device. However, it should be noted that, during the experimental sessions, the parameters normalized and, in the second part of the tests, in which the sanitation device was switched on, they were almost always within the range of acceptability. For this reason, it can be hypothesized that these small differences did not affect the IR results. It is known that some components of the HVAC system could be sources of microbial contamination and spreading. In particular, filters, which retain microorganisms, dust, and particulate matter, can be colonized by the microbial component, which can grow and be released from the filter, in favorable environmental conditions [33]. Furthermore, HVAC systems are regularly stopped to save energy. Throughout these periods of ventilation stoppage, the microbial populations collected by the filters are not exposed to airflow and, consequently, the microorganisms are less subject to desiccation phenomena. Thus, if certain conditions occur (such as humidity level, temperature and nutrients), microorganisms can grow significantly during these stoppages of HVAC systems and fungal spores in particular can germinate and grow [34,35]. Consequently, airborne pathogens may spread to other indoor spaces through air-conditioning return air ducts when they are reactivated. The CSA device proposed and evaluated in this study, with a filter exposed to UV-C LED irradiation, does not present these drawbacks. Previous studies have shown that the disinfection efficacy of in-duct UV-C lamps is increased by 20% when considering the duct wall reflection, compared to under non-reflection conditions [22]. In the CSA system, the reflecting surface inside the sub-ducts makes it possible to amplify the effect of the UV-C LEDs with a linear decrease in efficiency at the far ends (data not shown). Sanitation is considered a business topic in the COVID-19 era, having recorded a total turnover of 20 billion euros, for a counter-value of 500,000 employees in terms of workforce. Moreover, commercial and economic growth was followed by the consciousness of the need of low-environmental-impact techniques and technologies. Indeed, many chemical-based sanitation devices produce and release byproducts that are harmful and toxic to the environment. Other systems (i.e., ozonation- and UV-based) can generate ozone and mercury emissions. Furthermore, some systems require the replacement of exhausted components, particularly in the case of devices based on sanitation through microorganism retention (i.e., HEPA filters) or UV lamps, that have a shorter lifetime than LEDs. For these reasons, a sanitation system founded on energy-saving LED UV-C lamps (90% of the energy converted into UV-C rays) is an eco-friendly, ozone-free and sustainable solution with an extremely low impact on the environment.

## 5. Conclusions

In some trains, overcrowded environments in which a great percentage of the air is recycled, the placement of sanitation air systems able to maintain low levels of microorganisms and pollutants is essential. The device tested, thanks to the high efficiency it demonstrated at microbial inactivation, could be very useful in this regard and its use could be proposed in train settings. To the best of our knowledge, no data about the UV-C inactivation of aerosolized SARS-CoV-2 is yet available. However, recent studies demonstrated SARS-CoV-2’s susceptibility to UV-C [24,36]. The results of this study suggest the effectiveness of the CSA system at the inactivation of microorganisms other than those tested with lower inactivation UV doses, such as Coronavirus, included SARS-CoV-2 [24,37]. Additionally, optimal ventilation in railway vehicles and increasing the number of air exchanges per hour, as well as proper cleaning and maintenance of conditioning systems, may contribute to further reducing the risk of spreading aerodispersed microorganisms, including SARS-CoV-2. 

## Figures and Tables

**Table 1:** Microbial concentrations (CFU/m^3^ ± SD, average of three determinations) in air sampled downstream of the sanitation system in the test rig, measured at 1000–2000–3000 m^3^/h (ISO, 2019). Inactivation percentages (% IR ± SD) are reported.

Airflow Rates (m^3^/h)	1000			2000			3000		
	Off *	On *	% IR	Off *	On *	% IR	Off *	On *	% IR
***E. coli* ATCC25922**	5220 ± 11	36 ± 19	99.31 ± 0.5	4203 ± 55	36 ± 19	99.14 ± 0.4	4023 ± 145	42 ± 25	98.96 ± 0.6
***B. subtilis* ATCC6633**	971 ± 59	0	100	714 ± 92	0	100	533 ± 11	3 ± 2.78	99.44 ± 0.5
***Cladosporium* spp.**	1090 ± 21	333 ± 12	69.45 ± 0.5	875 ± 25	281 ± 30	67.89 ± 2.5	822 ± 11	264 ± 14	67.88 ± 1.3

* “off”: test performed with the sanitation system switched off and the ISO Coarse 90% filter removed; “on”: test performed with the sanitation system switched on and the ISO Coarse 90% filter installed. % IR: inactivation rate.

**Table 2:** *E. coli* concentrations (CFU/m^3^ ± SD) in air sampled downstream of the sanitation system in the train setting. Inactivation percentages (% IR ± SD) are reported.

Nebulization Time (Min)	2		
Sampling Sites	Off *	On *	% IR
**A**	364.00 ± 25.00	19.45 ± 2.75	94.58 ± 1.13
**B**	513.50 ± 39.50	19.45 ± 2.75	96.07 ± 1.07
**C**	475.00 ± 47.00	19.45 ± 8.35	95.69 ± 2.18
**D**	472.50 ± 83.50	38.85 ± 5.55	91.73 ± 0.29
**E**	453.00 ± 103.00	24.55 ± 13.45	93.57 ± 4.43
**F**	333.50 ± 44.50	22.20 ± 11.10	92.77 ± 4.29
**G**	350.00 ± 17.00	27.75 ± 5.55	92.13 ± 1.12
**H**	491.50 ± 97.50	27.80	94.11 ± 1.17

*”off”: test performed with the sanitation system switched off and the Coarse >90% filter removed; “on”: test performed with the sanitation system switched on and the Coarse >90% filter installed. % IR: inactivation rate. Sampling sites: proximal (**A**), central (**B**), and distal (**C**) air vent panels of the upper central section of the train car; the proximal (**D**), central (**E**), and distal (**F**) air vent panels of the lower central section of the train car; the proximal (**G**) and distal (**H**) air vent panels in the terminal section of the train car.

**Table 3:** UV-C dose (D) for each microbial species used in the sanitation experiments in the test rig, at each airflow rate (ISO, 2019).

Microbial Species	Airflow Rate (m^3^/h)	D (J/m^2^) *
***E. coli* ATCC25922**	1000	13.10
	2000	12.53
	3000	12.01
***B. subtilis* ATCC6633**	1000	n.d.
	2000	n.d.
	3000	30.73
***Cladosporium* spp.**	1000	564.66
	2000	540.89
	3000	540.85

* See Materials and Methods for the equation. n.d., not determined. It was not possible to calculate the D value, since the microbial concentration measured with the system “on” was “0”.
